# Acquisition of an obligate environmental symbiont may be limited in the arboreal environment

**DOI:** 10.1093/femsec/fiaf045

**Published:** 2025-04-25

**Authors:** Liam T Sullivan, Suzanne E Kelly, Alison Ravenscraft, Martha S Hunter

**Affiliations:** Graduate Interdisciplinary Program in Entomology and Insect Science, The University of Arizona, Tucson, AZ 85721, United States; Department of Entomology, The University of Arizona, Tucson, AZ 85721, United States; Department of Biology, The University of Texas at Arlington, Arlington, TX 85721, United States; Graduate Interdisciplinary Program in Entomology and Insect Science, The University of Arizona, Tucson, AZ 85721, United States; Department of Entomology, The University of Arizona, Tucson, AZ 85721, United States

**Keywords:** *Caballeronia*, environmental acquisition, gut symbionts, Hemiptera, host-symbiont interactions

## Abstract

Many eukaryotic organisms have environmentally acquired microbial symbionts. In animals, microbes commonly occupy the gut and may supply critical nutrients. The leaf-footed bug, *Leptoglossus zonatus* (Coreidae), is a true bug that is dependent upon ingestion of the free-living, soilborne bacterium *Caballeronia* early in development for growth and reproduction. In 2019 and 2020, we tested the ability of second instar *L. zonatus* to acquire *Caballeronia* in the canopy of pomegranate trees where *L. zonatus* are often found. We compared the acquisition rate of *Caballeronia* in nymphs left to forage for the symbiont to bugs fed *Caballeronia* in advance. Additionally, we aimed to determine whether the microhabitat of potential symbiont sources influenced acquisition success. We hypothesized that the acquisition rate would be heterogeneous among treatments. In 2019, ∼30% of experimental bugs acquired *Caballeronia*, compared to 75% of those fed the symbiont. In 2020, only about 4% of experimental bugs acquired any symbiont. The symbiont composition of caged bugs differed, and strain diversity was reduced relative to wild bugs. We concluded that *Caballeronia* is present in the canopy environment, but nymphs may fail to acquire it in the fragments of habitat represented by caged branches, suggesting a cost to host dependency on environmentally acquired symbionts.

## Teaser

An arboreal insect with an obligate, environmentally acquired bacterial symbiont was confined in its habitat to examine symbiont acquisition rate.

## Introduction

Microbial symbionts have played a fundamental role in the evolution of eukaryotes (Rosenberg et al. 2010, McFall-Ngai et al. 2013, Martin et al. 2017). Some symbionts, including many gut microbiota, are acquired from the host's environment. When a host is reliant upon these partners, symbiont acquisition is critical for survival. The dispersion and composition of microbial communities is heterogeneous and influenced by biotic and abiotic conditions (e.g. plants, pH, nutrients, moisture) (Fierer and Jackson 2006, Bryant et al. 2012, Chen et al. 2021). As a result, hosts dependent upon an environmentally acquired symbiont may risk failing to acquire a partner entirely.

In insects, symbionts required for host nutrition are commonly transmitted vertically from mother to offspring, either within or on the egg (Salem et al. 2015, Vorburger and Perlman 2018, Hosokawa and Fukatsu 2020), or horizontally within social colonies (e.g. Salem et al. 2015, Kwong and Moran 2016) with near perfect transmission between generations. In such cases, intimate partners have intertwined life histories and often are no longer able to survive without their host (McCutcheon and Moran 2012, Bennett and Moran 2013, 2015). In contrast, many facultative or commensal extracellular symbionts are free-living and are acquired from environmental reservoirs (e.g. soil) or other insects (Engel and Moran 2013, Ravenscraft et al. 2019). The focal species of this study, the arboreal leaf-footed bug, *Leptoglossus zonatus* (Dallas) (Hemiptera: Coreidae), belongs to one of seven families with a shared nutritional partner (Kikuchi et al. 2011a) soil-dwelling bacteria of the genus *Caballeronia* (Betaproteobacterium: Burkholderiaceae) (Kikuchi et al. 2007, 2011a, Kikuchi and Yumoto 2013, Takeshita and Kikuchi 2020). These symbiotic bacteria are ingested by the host in each generation and undergo strict selection within the insect midgut (Kikuchi et al. 2007, 2011b, Ohbayashi et al. 2015). *Caballeronia* symbionts colonize and proliferate within crypts of the midgut 4 region (M4). In the model system *Riptortus pedestris* (Alydidae), *Caballeronia* has been shown to recycle nitrogenous host waste and synthesize essential amino acids (Kikuchi et al. 2005, 2007, Ohbayashi et al. 2019). Bug hosts in the wild may contain one or more *Caballeronia* species (hereafter simply *Caballeronia)*. Other bacterial species from within the *Burkholderia sensu lato* and allied genera can also colonize the M4 gut region (Kikuchi et al. 2005, 2007, Itoh et al. 2019). In *R. pedestris*, however, these are generally outcompeted when *Caballeronia* is also present (Itoh et al. 2019). Though hosts are capable of filtering *Caballeronia* from a diverse soil environment, acquisition may still present a challenge in a spatially complex environment. Alternatively, if *Caballeronia* is abundant in the environment of nymphs that need to acquire it, e.g. in nearby soil, or on foliage where bugs release the symbiont in feces (Acevedo et al. 2021, Villa et al. 2023), there may be little risk of failure to find the symbiont.

*Leptoglossus zonatus* is an arboreal species with a native range extending from the western United States through South America (Buss et al. 2005) and is a common pest of orchard crops. In the southwestern USA, *L. zonatus* feeds on orchard crops like pomegranates, almonds, citrus (Xiao and Fadamiro 2009, Joyce et al. 2019), or native trees like desert willow (Jones 1993). In contrast, *Caballeronia* (formerly the SBE clade of *Burkholderia)* are widespread free-living soil bacteria known to associate with insects and plants. In pomegranate orchards, individual *L. zonatus* tend to host 1–5 species of *Caballeronia*, with one species being dominant and the additional ones present at much lower abundance, if at all. The array of *Caballeronia* species found in *L. zonatus* adults is largely a subset of taxa in the Burkholderiaceae found in the soil below the trees (Ravenscraft et al. 2024). Given the prevalence and occasional pest status of *L. zonatus*, one might hypothesize that *Caballeronia* is ubiquitous in the environment and not limiting for nymphs. Eggs are laid in chains, often on the underside of host plant leaves (Fernandes et al. 2015). Hatching in the canopy, nymphs become able to ingest the symbiont during their second instar, and must acquire *Caballeronia* or most bugs fail to reach adulthood (Hunter et al. 2022). This leaves the question: is *Caballeronia* available in the canopy, or does the nymph have to travel a greater distance, possibly to the soil, for acquisition to occur?

Symbiont transmission may occur through environmental means, dispersed onto the phyllosphere through wind and rain. Alternatively, the symbiont may be acquired horizontally from adult feces. Under laboratory conditions, both *L. zonatus* (LTS *et al*. unpubl. data) and another coreid squash bug, *Anasa tristis* (DeGeer) , can acquire the symbiont from conspecific feces (Villa et al. 2023). The role of fecal transmission under field conditions is unclear. A host may not deposit feces in an optimal location for bacterial survival and growth, e.g. on plant surfaces or in adequate abundance for acquisition to occur. The environmental conditions may further complicate symbiont persistence, and may shape symbiont identity within hosts if strains are differentially heat- or dessication-resistant. Unfavorable conditions (high temperatures, low humidity, and solar radiation) could render symbiont-laden feces an ephemeral resource for searching nymphs, hinder the survivorship of microbes in the phyllosphere, or simply increase mortality of bugs, reducing the amount of feces available to nymphs. While we know the symbiont is vital for *L. zonatus* survival, it is not yet clear how often these arboreal bugs fail to acquire *Caballeronia*.

The current study aimed to determine if *Caballeronia* can be acquired by early instar *L. zonatus* nymphs in the canopy, where second instar nymphs are found. To address this, we confined aposymbiotic, lab-reared *L. zonatus* nymphs in cages on branches of pomegranate trees under various treatment conditions intended to assess the availability of the *Caballeronia* symbiont in the tree canopy from potential horizontal or environmental sources, i.e. the phyllosphere, potentially contaminated fruit, or conspecific adults. We hypothesized that the symbiont acquisition rate would be heterogeneous among experimental treatments.

## Methods

**Figure 1.**
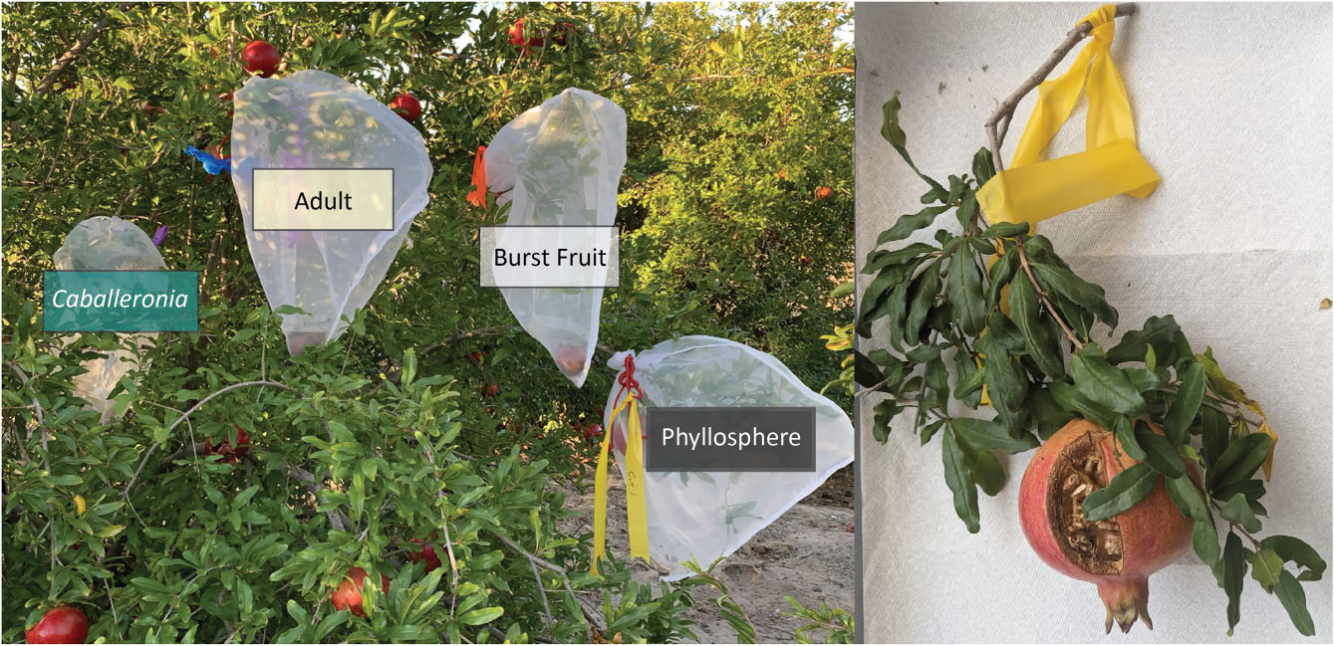
Field cages used in 2019 (L), with cages labeled with colored flagging tape designating treatment. (R) Example of enclosed foliage and fruit in each cage, at harvest. The cage shown is from the “Cut” treatment.

*Field cage study*: Field cages were placed in pomegranate trees (*Punica granatum)* at the University of Arizona (UA) West Campus Agricultural Center in Tucson, AZ, USA (32.258, −111.003) in September 2019 and October 2020. In each year, on each of 10 trees, one cage was attached for each of the four (2019) or three (2020) treatments (descriptions below). Fabric mesh cages (406 × 406 mm) were secured over ∼350 cm branch tips that bore a pomegranate fruit (Fig. 1). Twelve lab-reared early second instar *L. zonatus* nymphs were added to each cage according to treatment.

*Experimental treatments*: All cages enclosed a pomegranate branch and included a single pomegranate fruit. In all cages, the pomegranate had either burst open naturally (see Burst Fruit treatment) or a wedge was removed (Fig. 1) to expose seeds, providing similar access to this food source. In 2019, nymphs were assigned to one of four treatments, designed to distinguish among potential sources of *Caballeronia*. Treatments included the *Caballeronia*-fed control (“*Caballeronia*”). Bugs in all treatments aside from the *Caballeronia* treatment were aposymbiotic at the outset of the experiment. In a second treatment (“Burst Fruit”), nymphs were enclosed with a pomegranate fruit that had opened naturally, exposing the seeds. Not limited to pomegranates, fruit “splitting” or “cracking” is a common issue in cultivation where the fruit bursts opens naturally, exposing the flesh and seeds, e.g. Singh et al. (2020). We had observed aggregations of bugs within burst fruits in the orchard and hypothesized that the fruits may serve as a source of horizontal transmission if previously visited by *L. zonatus*. A potentially more direct source of horizontal transmission was provided in the third treatment by the placement of a single adult male present in the cage (“Adult”), collected from another tree within the orchard at the time nymphs were introduced. We chose males because they would not lay eggs in the cages. In the last treatment, nymphs were confined without any potential sources of the symbiont other than leaf surfaces where bacteria may have been deposited by wind or insects (“Phyllosphere”). We considered that these nymphs were least likely to encounter *Caballeronia* within the cage. In 2020, the experiment was repeated with three treatments, (i) the *Caballeronia* treatment; (ii) the Phyllosphere treatment; and (iii) a modified “Adult” treatment using a single, lab-reared virgin female in case the low rate of horizontal transmission of *Caballeronia* from males in 2019 was sex specific. A laboratory-reared virgin female was used in the 2020 “Adult” treatment to prevent fertile eggs from being laid within the cages. Since no *L. zonatus* reached a healthy adult stage without *Caballeronia* in the field (Ravenscraft et al. 2024) or the laboratory (Hunter et al. 2022), all adults placed in cages in 2019 and 2020 were presumed to be symbiotic.

In each year, a subset of the *Caballeronia* group cages were visually inspected every few days to determine developmental stage. When 80% or more of the bugs within these cages reached adulthood, at ∼5 weeks, each cage with enclosed *L. zonatus* and foliage were clipped and brought to the laboratory (Fig. 1). The life stages of surviving bugs and the mortality of individuals were recorded. All surviving insects were frozen at −80°C. A subset of insects were used for diagnostic PCR to assess symbiont acquisition, up to seven individuals per cage when available, with a minimum of two insects per juvenile instar and two adults per sex. Any cages with more than 12 bugs, indicating wild bugs had been enclosed within or entered the cage during the experiment, were excluded from analysis.

*Insect rearing: Leptoglossus zonatus* were reared in the laboratory at the UA at 27°C, 16:8 L/D on potted cowpea (*Vigna unguiculata*) plants and fed raw peanuts (Hunter et al. 2022). Eggs were collected at 24h intervals to synchronize emergence and development. After hatching, Petri dishes of first instar nymphs were given deionized water with 0.05% ascorbic acid (DWA) in 2 ml vials with cotton plugs. Water was withheld from all dishes for 24h prior to symbiont feeding to encourage ingestion of the symbiont. Bugs in the *Caballeronia* (control) treatment were fed ∼2.5 ml of *Caballeronia* (strain Lz049) in suspension on nonsterile cotton gauze for 24h (see culture methods below). All other treatment bugs were given DWA during this period, thus remaining aposymbiotic before release. Following the 24h period, nymphs were introduced into field cages according to treatment.

*Bacterial culturing*: Strain Lz049 is an isolate of *Caballeronia* in the Coreoidea subclade, which currently contains no named species (Ohbayashi et al. 2022). It was isolated from a single *L. zonatus* adult collected in Tucson, Arizona in 2018 from the UA West Campus Agricultural Center. The insect's M4 organ was aseptically removed, rinsed in sterile PBS, and incubated on a shaker at ambient temperature in yeast-glucose (YG) broth (Kikuchi et al. 2011a). This organ culture step improves the recovery of the symbiont (Xu et al. 2016a). After 24h, the M4 was removed from the broth, homogenized with a sterile test tube pestle, and plated on YG agar. We isolated a colony and identified it as *Caballeronia* via bidirectional Sanger sequencing of the 16S rRNA gene using the universal bacterial 16S primers 10F/1502R (Xu et al. 2016b). The isolate was stored at −80°C in a 25% glycerol stock (Kikuchi et al. 2011a).

Bacterial culturing and insect feeding followed methods developed by Kikuchi et al. (2007, 2011a). For feedings, bacteria were grown on YG media (Kikuchi et al. 2011a) at room temperature (∼18–20°C) and liquid YG media was inoculated from single colonies. Liquid culture was grown at room temperature on a rotary shaker (280 rpm) overnight. Prior to presentation to nymphs, cells were washed in sterile DI water and resuspended in DI water to ∼5.0 × 10^9^ CFU/ml.

*Dissection, DNA extraction, and diagnostic PCR*: Insects were prepared for symbiont DNA extraction using one of two methods. In one, a portion of the midgut 4 region (M4) was removed, placed into 1.5 ml tubes containing 5%–10% Chelex 100 (MilliporeSigma) solution and ground using a pipette tip. In the second, abdomens were homogenized in 800 µl of sterile deionized water with 3.2 mm chrome steel beads at 4 m/s for 80 s. Ten µl of homogenate was transferred into 0.5 µl tubes with 5%–10% Chelex 100 solution. For both methods, 8 µl of proteinase K (20 mg/ml) was added and tubes were incubated overnight at 37°C. Diagnostic PCR was performed using primers Burk16SF (5′-TTTTGGACAATGGGGGCAAC-3′) and Burk16SR (5′-GCTCTTGCGTAGCAACTAAG-3′) designed to amplify *Caballeronia* and closely allied Burkholderiaceae species (Kikuchi et al. 2005), (95°C for 10 min, 30 cycles of 95°C for 30 s, 55°C for 1 min, and 72°C for 1 min). PCR products were visualized on an agarose gel with SYBR Green (Hunter et al. 2022).

*Microbiome analysis*: Samples positive for *Caballeronia* and allies, excluding those from the *Caballeronia* treatment, underwent Illumina amplicon sequencing at University of Texas Arlington (UTA) with *Burkholderia sensu lato-*specific 16S rRNA primers (Tago et al. 2014) (Bf: 5′-TAGCCCTGCGAAAGCCG-3′ and Br: 5′-GCCAGTCACCAATGCAG-3′) to characterize symbiont strain diversity. We hypothesized that the symbiont diversity of caged *L. zonatus* would be reduced relative to free-roaming wild bugs collected in the orchard. Library preparation and data processing methods followed (Ravenscraft et al. 2024). Briefly, the *Burkholderia s. l*. 16S rRNA gene was amplified using Illumina's two-step library preparation approach (Illumina Inc. 2013). PCR blanks produced no bands during gel electrophoresis and were therefore excluded from sequencing. An equal mass of each sample was bidirectionally sequenced on 600 cycle paired-end runs on an Illumina Mi-Seq platform at the UTA Life Science Core Facility. Sequences were combined with data from wild insects collected in September and October of 2019 and 2020 from the same site (Ravenscraft et al. 2024). We filtered poor quality reads and inferred bacterial amplicon sequence variants (ASVs, which approximate bacterial strains) [DADA2, (Callahan et al. 2016)]. To assign ASVs to subclades within the *Burkholderia s.l*., we used SEPP implemented in QIIME2 (Mirarab et al. 2012, Bolyen et al. 2019) to place them onto a reference phylogeny (Ravenscraft et al. 2024). Data were rarefied to 9123 reads per sample, where sample rarefaction curves plateaued. Finally, we built a *Caballeronia* phylogeny (see below) and used it to agglomerate ASVs into lineages at a cophenetic distance of 0.1 [*phyloseq*, (McMurdie and Holmes 2013)], which approximates *Caballeronia* species (Fig. 5).

To assign ASVs to lineages and subclades of *Caballeronia*, we placed them onto a reference phylogeny built by Ravenscraft et al. (2024). Briefly, the reference tree was built using five genes (rpoB, rpoC, rplA, recA, and 16S rRNA) extracted from *Burkholderia sensu lato* genomes downloaded from NCBI and the Integrated Microbial Genomes database, supplemented with additional 16S sequences from our own *Caballeronia* isolate library. Sequences were aligned with MAFFT (Katoh and Standley 2013) and a maximum-likelihood tree was built using RAxML with the GTR+Γ model of nucleotide substitution (Stamatakis 2014), with separate partitions for each codon position of each protein coding gene plus one partition for the 16S rRNA. Node support values were calculated using rapid bootstrapping which was halted automatically based on the MRE criterion. We then placed ASVs onto the resulting phylogeny using SEPP implemented in QIIME2 (Mirarab et al. 2012, Bolyen et al. 2019). The *tip_glom* function (*phyloseq)* was used to agglomerate sequence variants into lineages with a cophenetic distance of 0.1 (McMurdie and Holmes 2013), which equates very roughly to the species level.

*Climate data*: Temperature and relative humidity (%RH) were logged for the duration of the experiment each year using a HOBO MX2401 data logger (Onset Brands). In 2019, readings were taken at 5-min intervals and reduced to hourly intervals in 2020. The logger was placed in a pomegranate tree ∼50 cm above the soil in a central location within the orchard.

*Statistical analyses*: All analyses were performed in R version 4.4.0 (R Core Team 2024).

Surviving insects were categorized as “early” (third – fourth instar) or “late” (fifth instar – adult). We analyzed the (i) proportion of bugs in late life stages and (ii) proportion of *Caballeronia-*positive individuals, using a logistic regression using a quasibinomial distribution [*lme4* (Bates et al. 2015)]. The number of dead within treatment was assessed using a generalized linear model with Poisson errors [*lme4* (Bates et al. 2015)]. Model effects were assessed using the likelihood ratio test (*drop1*). Post-hoc comparisons were made using Tukey's HSD [*multcomp* (Hothorn et al. 2008)].

Average daily high for temperature and %RH were compared between years using a Mann-Whitney U test.

To compare the symbiont diversity of caged versus wild insects, we conducted a permutation test. In each of 10,000 iterations, we randomly selected one insect from each cage (since observations within cages are not independent). We calculated the difference in total symbiont richness hosted by these 19 bugs versus richness in 29 wild bugs collected in the orchard at the same time. To estimate the expected difference under the null hypothesis, insect origin (caged or wild) was randomized among the 48 bugs and the richness difference was recalculated. The *P*-value represented the proportion of instances in which the difference in richness between the groups was less than or equal to the difference under the null expectation richness, multiplying by two to account for a two-tailed test.

## Results

**Figure 2.**
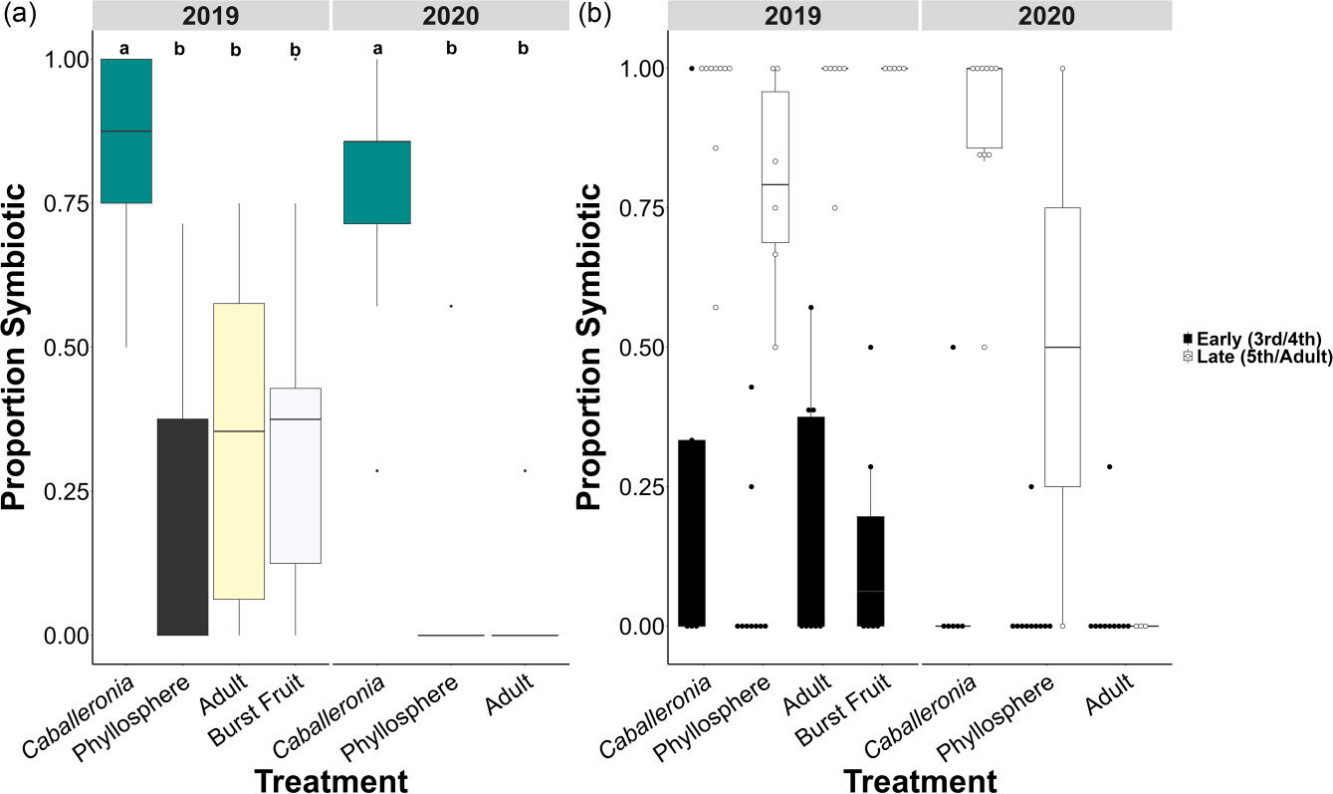
Field cage experiment in 2019 and 2020. (a) Boxes represent the proportion of symbiont positive bugs in each replicate by treatment. Lower case letters reflect pairwise comparisons between experimental treatments. (b) Boxes represent proportion of symbiont positive bugs in different life stage groups, either “early” (third or fourth instar) or “late” (fifth instar or adult). Dots represent outliers.

Caballeronia *acquisition*: The proportion of bugs that were colonized by *Caballeronia* varied significantly by treatment in 2019 (*F* = 6.62, *P* = .001) and 2020 (*F* = 34.94, *P* = 3.23 × 10^−8^; Fig. 2a). Insects in the *Caballeronia* treatment had higher rates of *Caballeronia* colonization than in any other treatment. Relatively few individuals acquired the symbiont in the other treatments in 2019, and almost no insects acquired a symbiont in any treatments other than the *Caballeronia* treatment in 2020 (Fig. 2a). Pairwise comparisons revealed no differences among the treatments that were aposymbiotic at the outset in either year (Fig. 2a). *Caballeronia* acquisition was further associated with development to the later development stages over the course of the experiment (Fig. 2b).

**Figure 3.**
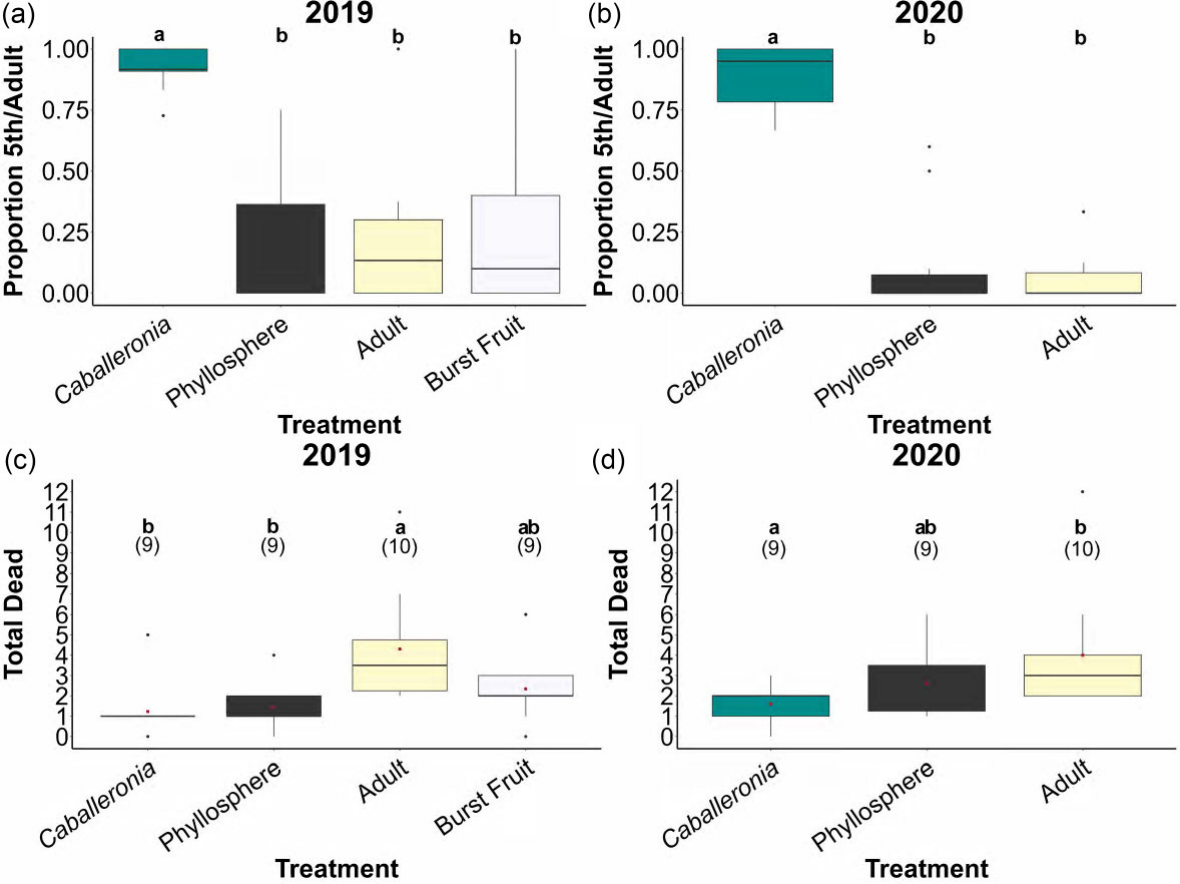
Developmental stage and mortality of *L. zonatus* in 2019 (a) and 2020 (b). In 2019 (a) and 2020 (b), boxes represent the proportion of bugs that reached the fifth instar or adult stage in each replicate. Lower case letters reflect pairwise comparisons between each experimental treatment for developmental stage. Different letters reflect statistically significant differences. Mortality within each replicate across treatments in 2019 (c) and 2020 (d). Numbers above boxes represent the number of replicate cages included in each group. Red dots represent mean dead within treatment. Lower case letters represent pairwise comparisons. In all figures black dots represent outliers.

*Insect development*: The proportion of bugs that developed to the late life stages varied significantly by treatment in 2019 (*F* =9.31 , *P* = .001) and 2020 (*F* = 4.36, *P* =6.36×10^−08^), showing a large difference between the *Caballeronia* and the other treatments (Fig. 3a, b). Pairwise comparisons showed no differences in the life stage reached among the treatments in which the bugs were aposymbiotic at the outset in either year (Fig. 3a, b).

*Mortality*: Overall mortality was similarly low across treatments in both years. While treatment had a significant effect on mortality (2019: LRT = 22.51, *P* = 5.1x10^-5^, 2020: LRT = 10.725, *P*= 0.005), only mortality in the “Adult” treatments (Fig. 3c, d) showed significantly greater mortality rate than the *Caballeronia* treatments in both years.

**Figure 4.**
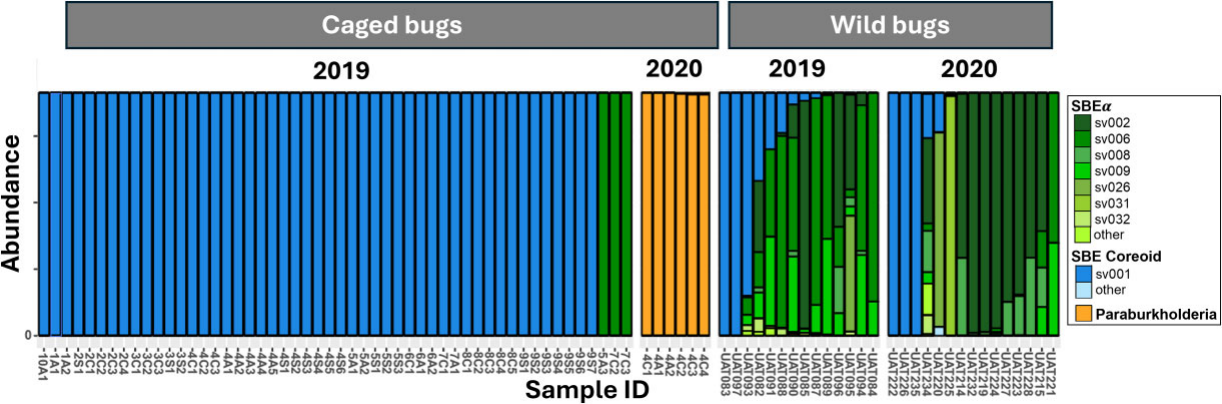
Relative abundances of *Burkholderia s. l*. lineages within each insect. Bars represent individual insects and colors indicate relative abundances of the lineages within the amplicon data. Bugs are sorted by the abundance of the dominant lineages. “Caged” samples represent all bugs reported positive for *Caballeronia* in one of 17 (2019) or 2 (2020) cages and categorized according to *Caballeronia* (= “SBE”) subclades (Ohbayashi et al. 2022). “Wild” samples are individual bugs from Ravenscraft et al. (2024), collected at the same field site at the same time.

**Figure 5.**
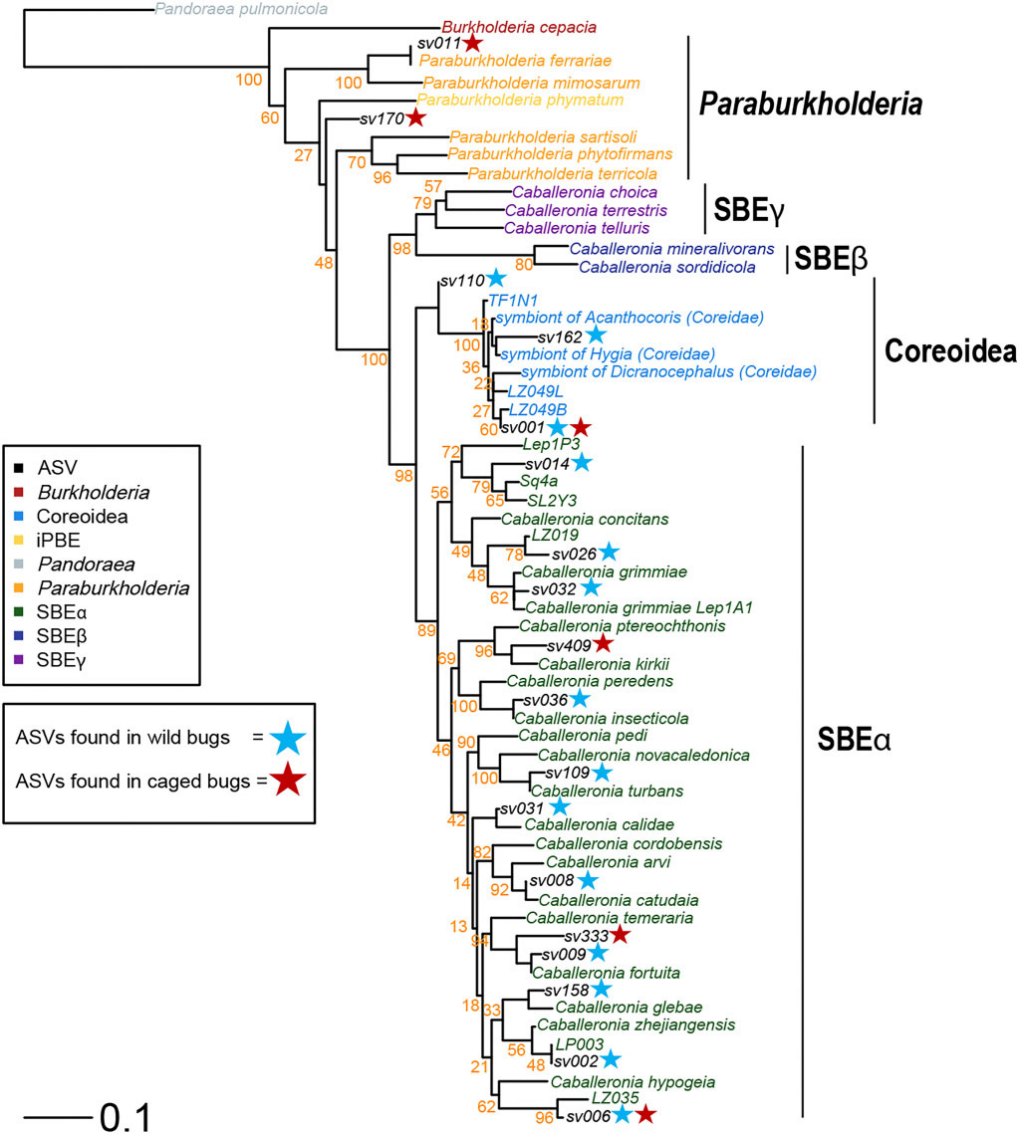
Phylogenetic placement of the Illumina amplicon lineages from this study (in black text). Reference sequences are colored based on their taxonomic clade. Reference sequences that did not have closely related SV lineages were dropped from the figure for improved readability. Bootstrap values (orange) are reported for the reference tree. The tree was rooted with *Pandoraea pulmonica*. Scale bar indicates 0.1 nucleotide substitutions per site.

Caballeronia *composition*: The symbiont community of caged bugs was significantly less diverse than that of wild bugs (permutation test: *P* = 0.038). We detected a total of six *Burkholderia s. l*. lineages in 58 caged bugs representing 17 cages in 2019 and two cages in 2020. In caged bugs, three dominant lineages accounted for most reads, compared to eight dominant lineages (14 total) in the 29 wild bugs (Fig. 4). In wild bugs from both years, 24% of symbiont sequences belonged to the Coreoidea-associated *Caballeronia* clade and 76% belonged to *Caballeronia* clade SBEα (Ohbayashi et al. 2022) (Figs 4, 5). In contrast, the 52 caged insects in 2019 hosted 94% Coreoidea-associated and 6% SBEα, while in 2020, the six caged insects recovered hosted 99.7% *Paraburkholderia* (Fig. 5).

*Temperature and humidity*: Daily high temperatures were significantly hotter and drier in 2020 compared to 2019 (W = 212, *P* = 6.27×10^−05^). Median high temperatures in 2019 were significantly lower at 34.10°C compared to 38.14°C in 2020 (Fig. S1). The median daily high humidity was significantly higher in 2019 (75.00% RH) than in 2020 (37.98% RH; W = 1005, *P* = 2.88×10^−11^; SI Fig. 2).

## Discussion

*Symbiont acquisition is limited in the canopy*: When restricted to a unit of their customary habitat in the canopy, *L. zonatus* nymphs were unlikely to acquire their obligate symbiont. In both years, few experimental bugs acquired *Caballeronia* in cages in the field, only ∼30% in 2019 and none in 2020, although a few individuals acquired the related *Paraburkholderia* in 2020 (Figs 2a, 4). Interestingly, the treatments other than *Caballeronia* were equivalent in their rate of *Caballeronia* acquisition. This suggests that incorporating adult bugs and burst fruit aggregation sites within cages did not differentially enrich the habitat for *Caballeronia* as hypothesized, despite previously finding that frass is a potentially important source of viable *Caballeronia* in *L. zonatus* (LTS et al. unpubl data) and in another coreid (Villa et al. 2023).

*Host development tied to symbiont acquisition*: We observed that treatment influenced both the proportion symbiotic and the developmental stage at the end of the experiment (Fig. 2a, b). Most older stage bugs had acquired *Caballeronia*, while most bugs with delayed development, those in the fourth instar or earlier, were aposymbiotic (Fig. 2b). The results are congruent with the results of Hunter et al. (2022), in which remaining aposymbiotic significantly increased development time. Within a bug cohort, then, developmental stage may be a good proxy for symbiont status. While mortality was largely low and similar across treatments (Fig. 3c, d), the developmental delay associated with remaining aposymbiotic (Fig. 2b) demonstrates the cost of deferred symbiont acquisition. Even if nymphs could acquire the symbiont throughout their lives, slow-developing individuals that experience a delay in acquisition would experience decreased reproductive fitness (Nylin and Gotthard 1998). In addition, evidence from *R. pedestris* suggests the window for potentially acquiring the symbiont is likely to close before adulthood, making acquisition of the symbiont a race against time (Kikuchi et al. 2011b).

*Caballeronia acquisition rates may be driven by host density and environmental conditions*: While symbiont acquisition was low in 2019, it was almost entirely absent in 2020. While we can only speculate on the difference between the years, the higher daily temperatures and lower humidity of 2020 (Figs S1, S2) may have decreased bacterial survivorship in the phyllosphere. Further, relatively few wild *L. zonatus* were observed in 2020 compared to 2019, suggesting there were fewer adults excreting *Caballeronia* into the canopy. Together, the greater heat, aridity, and low bug density likely reduced symbiont availability. Possibly, the abiotic factors that may have reduced acquisition success in cages may have similarly impacted acquisition in wild *L. zonatus*, limiting the growth of the wild population.

*Symbiont diversity reduced in experimental nymphs: Caballeronia* diversity was reduced in caged bugs confined to the canopy compared to wild, free-roaming insects in the same orchard. The two lineages that colonized caged nymphs in 2019 represented less than one third of the *Caballeronia* diversity (14 lineages) in wild bugs (Ravenscraft et al. 2024). In 2020, the pattern was more striking; none of the four lineages that colonized experimental nymphs were detected in wild bugs or soil samples from the orchard (Ravenscraft et al. 2024) (Fig. 4).

At the clade level, the Coreoidea-associated group was much more prevalent in caged bugs than wild bugs (Fig. 4), suggesting this clade might be more available or competitive in the canopy compared to SBEα. Insect fitness outcomes for these subclades have not been compared; this will be a fruitful avenue for future work. Fascinatingly, caged bugs in 2020 were primarily colonized by bacteria of the genus *Paraburkholderia*, a primarily soil-dwelling group closely related to *Caballeronia*. While *Paraburkholderia* are rarely observed in Coreidae (Ravenscraft et al. 2024), members of the *Paraburkholderia* subclade, called the iPBE (insect-associated, plant beneficial and environmental) clade is associated with members of the family Largidae (Hemiptera) (Takeshita et al. 2015, Takeshita and Kikuchi 2020). Furthermore, the detected *Paraburkholderia* are outside of the iPBE clade. These results indicate that when it has no better alternative, *L. zonatus* can acquire *Paraburkholderia*, and this may allow nymphs to advance to adulthood. In the model *R. pedestris* (Fabricius), Itoh et al. (2019) found that non-*Caballeronia* members of the Burkholderiaceae (*Paraburkholderia* and *Pandoraea*) could colonize the bug gut; these conferred some benefits relative to remaining aposymbiotic but were inferior to *Caballeronia* for bug performance and were competitively excluded when *Caballeronia* was present (Itoh et al. 2019).

The low rate of *Caballeronia* acquisition, reduced symbiont diversity, and altered composition found in the caged bugs may be explained in a few ways. By confining nymphs, we may have prohibited them from reaching the soil where *Caballeronia* in pomegranate orchards is found (Ravenscraft et al. 2024). We expect the soil to be the primary habitat of *Caballeronia* and members of *Burkholderia sensu lato* (e.g. Kikuchi et al. 2005, 2007, 2011a). The soil may be close by for bugs that live on forbs like *R. pedestris* (Kikuchi et al. 2007, Lim 2013) or *Anasa tristis*. However, for arboreal bugs like *L. zonatus*, reaching the soil, ingesting the symbiont, and returning to the canopy would seem an arduous trek. It is unclear how successful *L. zonatus* are at finding *Caballeronia* in nature, even when the journey can be made. The catastrophic fitness deficits of remaining aposymbiotic suggests unsuccessful bugs will likely be absent from sampling efforts.

Conspecific feces might provide an alternative source of *Caballeronia* (Ravenscraft et al. 2020, Villa et al. 2023), but we did not see evidence that an adult in the cage significantly promoted symbiont acquisition (Fig. 2a). It is possible that the conditions of this experiment could have restricted access to this fecal resource. A single adult may not have provided a sufficient inoculum for the nymphs to find and ingest, even after weeks of cohabitation. Bugs in the coreoid and lygaeoid superfamilies are known to aggregate (Numata et al. 1990, Mitchell 2006, Addesso et al. 2012), but we do not know if there are critical mixes of stages or sexes, particular density thresholds, or behaviors that occur in aggregations that might facilitate transmission of the symbiont by nymphs. If there is a threshold in aggregation size for horizontal transmission to occur, we might predict positive density dependence for population growth, with explosive growth in seasons where conditions favor crowding, a concentration of symbiont-containing frass and easy symbiont acquisition by the next generation.

In nature, nymphal *L. zonatus* could disperse widely to acquire *Caballeronia* from various sources. While dispersal may solve the problem of acquisition, there are risks; movement away from the egg clutch is energy-intensive and may present risks for predation, starvation, and desiccation. Predation risks might be amplified for nymphs moving away from the natal “herd” of early instar nymphs that form after hatching, where they are presumably somewhat protected by their numbers (Lehtonen and Jaatinen 2016). Starvation could occur if these seed and fruit-feeding *L. zonatus* are unable to relocate fruits in the canopy after location and ingestion of *Caballeronia* some distance away from food. Desiccation might be a risk if nymphs are off the tree in pursuit of the symbiont and do not have access to plant sap as a water source (Mitchell 2006). We expect free-roaming bugs to pursue ingestion of *Caballeronia* in some way, given that aposymbiotic bugs do not reproduce.

Future work should test the hypothesis that the need to find *Caballeronia* may be limiting for arboreal coreid juvenile performance even when they are free to roam. Arboreal species dependent on *Caballeronia* are relatively common, at least within Coreoidea, and include species such as the giant mesquite bug, *Thasus neocalifornicus* (Bravilovsky & Barrera), and the invasive western conifer seed bug, *L. occidentalis* (Olivier-Espejel et al. 2011, Ohbayashi et al. 2022). Whether these species typically show a life history with higher rates of juvenile mortality due to constrained availability of the symbiont relative to their forb-feeding relatives would be interesting to explore. (Kikuchi et al. 2007, Kikuchi and Yumoto 2013, Hunter et al. 2022) Perhaps these potential limitations are largely overcome through horizontal transmission of *Caballeronia* in aggregations of adults and nymphs, or female preference for oviposition near areas of local enrichment of the symbiont. It is not yet clear how the distribution of symbiont in the phyllosphere or soil environment may influence the behavior of adult bugs. In sum, our results reveal a potential limitation to environmental acquisition of an essential symbiont and confirm more broadly that the spatial distribution of microbial partners may have critical effects on the behavior and fitness of animals that depend on them.

## Supplementary Material

fiaf045_Supplemental_File

## Data Availability

All data and scripts used in this study are available from Dryad data repository https://doi.org/10.5061/dryad.g4f4qrg0b. Sequence data is available through the Sequence Read Archive PRJNA1173607.
