# Supplementary material for: Acquisition of an obligate environmental symbiont may be limited in the arboreal environment
Source: FEMS Microbiol Ecol. 2025 Apr 25;101(5):fiaf045. doi: 10.1093/femsec/fiaf045 (PMC12063585; doi:10.1093/femsec/fiaf045)
Supplement: fiaf045_Supplemental_File [file fiaf045_supplemental_file.docx]

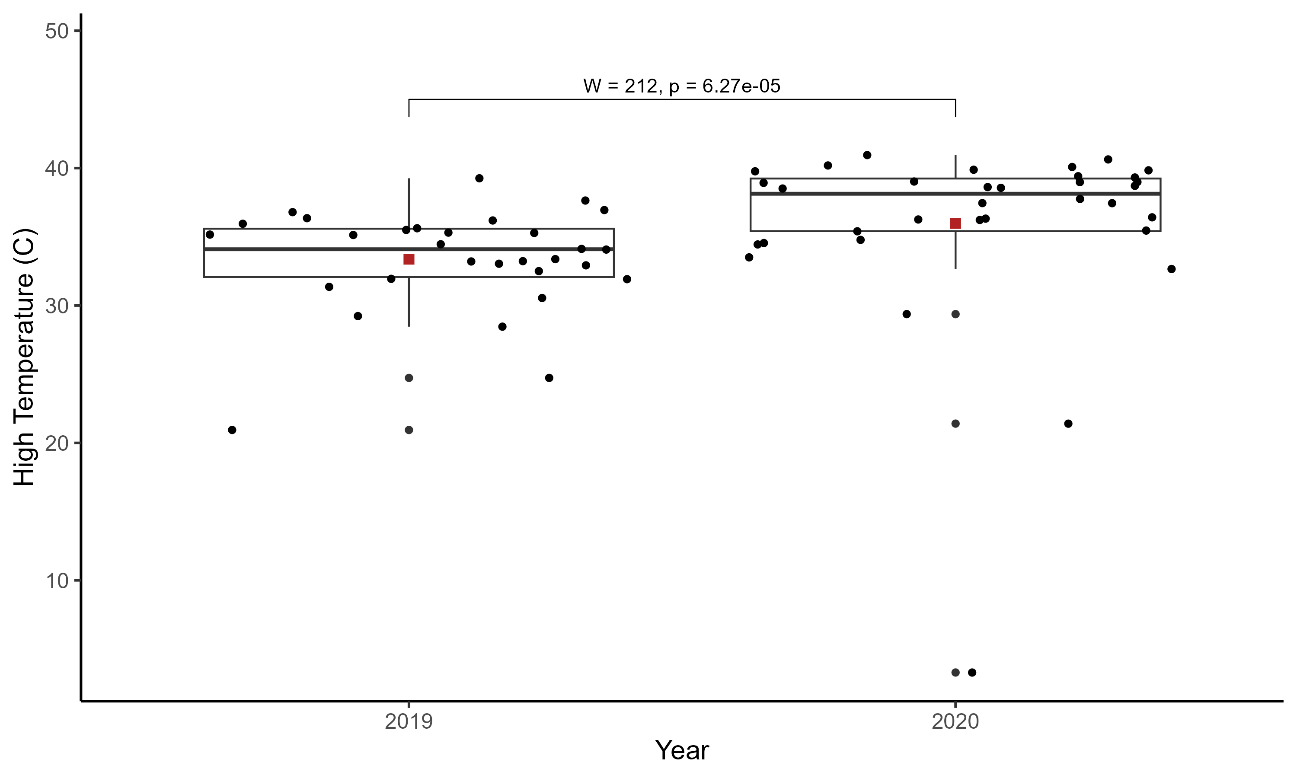


Figure 1. Daytime high temperature in each year. Red squares present mean high within each year.


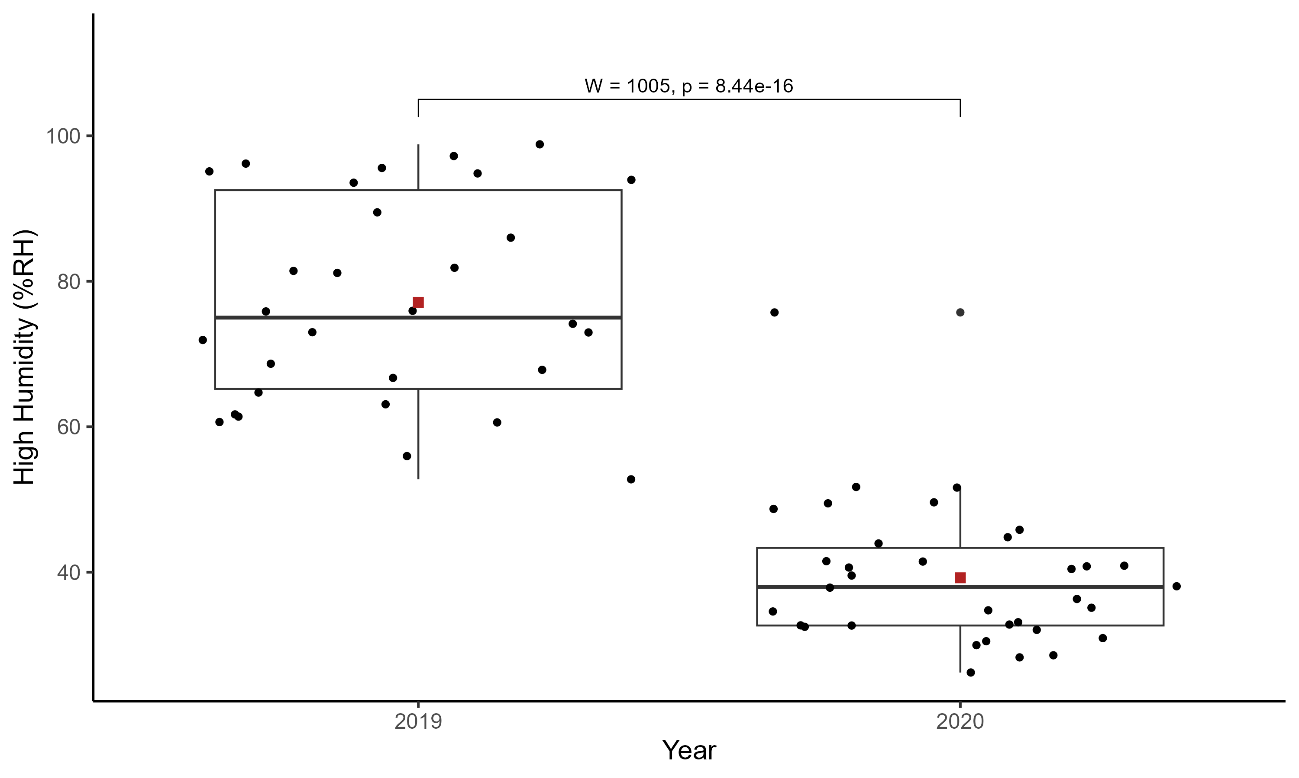


Figure 2. Daytime high humidity (%RH) in each year. Red squares represent mean high humidity within each year.


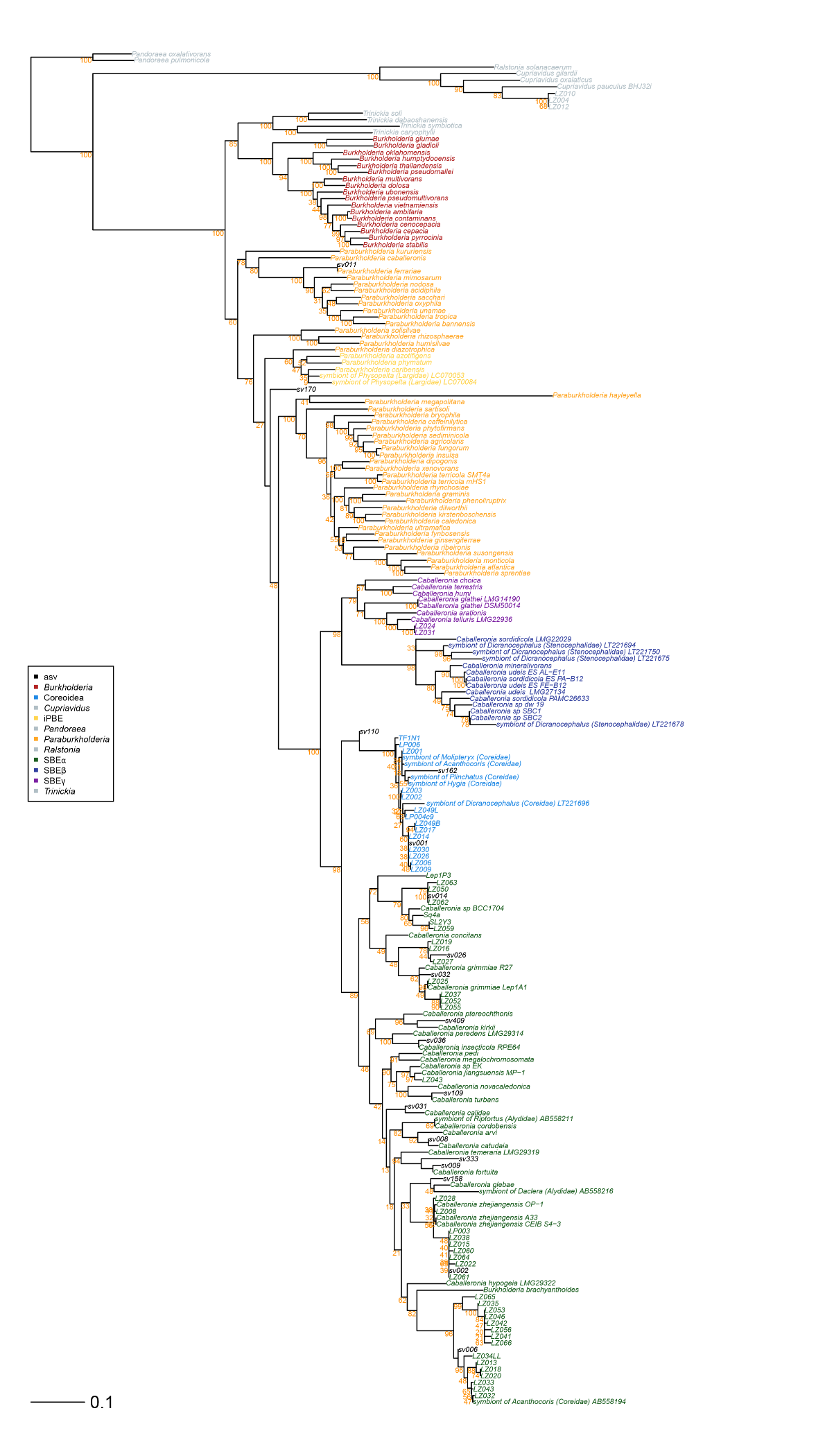


Figure 3. Phylogenetic placement of the Illumina amplicon lineages from this study (in black text). Reference sequences are colored based on their taxonomic clade. The tree was rooted with *Pandoraea* *pulmonica*. Scale bar indicates 0.1 nucleotide substitutions per site.
